# Decreased Physical Activity Among Youth Resulting From COVID-19 Pandemic–Related School Closures: Natural Experimental Study

**DOI:** 10.2196/35854

**Published:** 2022-04-15

**Authors:** Amanda Grimes, Joseph S Lightner, Katlyn Eighmy, Chelsea Steel, Robin P Shook, Jordan Carlson

**Affiliations:** 1 School of Nursing and Health Studies University of Missouri-Kansas City Kansas City, MO United States; 2 Department of Population Health University of Kansas Medical Center Kansas City, KS United States; 3 Center for Children’s Healthy Lifestyles & Nutrition Children’s Mercy Kansas City Kansas City, MO United States

**Keywords:** intervention, physical activity, nutrition, adolescents, formative research, COVID-19, pandemic, school closure, children, youth

## Abstract

**Background:**

The COVID-19 pandemic has resulted in the closure of schools and may have inadvertently resulted in decreased physical activity for youth. Emerging evidence suggests that school closures due to the COVID-19 pandemic could have hastened the inactivity of youth, possibly due to a lack of structure outside of school and increased access to sedentary activities.

**Objective:**

The purpose of this study was to assess changes in physical activity from pre–school closure (before the pandemic) to post–school closure (during the pandemic) among youth in spring 2020.

**Methods:**

This study used a natural experimental design; youth were enrolled in a physical activity study prior to the lockdown, which was enforced due to the pandemic. The number of device-assessed steps per day and moderate-to-vigorous physical activity minutes per week were measured by using a Garmin Vivofit 4 (Garmin Ltd) accelerometer over 8 weeks. Mixed effects models were used to compare physical activity variables, which were measured before and during the COVID-19 pandemic.

**Results:**

Youth were primarily Hispanic or Latinx (8/17, 47%) and female (10/17, 59%). The number of daily steps decreased by 45.4% during the school closure, from a pre–school closure mean of 8003 steps per day to a post–school closure mean of 4366 steps per day. Daily moderate-to-vigorous physical activity decreased by 42.5%, from a pre–school closure mean of 80.18 minutes per week to a post–school closure mean of 46.13 minutes per week.

**Conclusions:**

Youth are engaging in roughly half as much physical activity during the school closure as they were prior to the school closure. If additional evidence supports these claims, interventions are needed to support youths’ engagement in physical activity in the Midwest.

## Introduction

The lack of youth physical activity (PA) is a pervasive public health issue. Less than one-quarter of youths aged 6 to 17 years in the United States engage in the recommended 60 minutes of moderate-to-vigorous PA (MVPA) per day [1,2]. Regular PA can improve children’s physical health [2] and cognitive performances [3,4]. Physical inactivity can result in being overweight or obese [3] and increase one’s risk for cardiovascular disease [5,6] and type 2 diabetes [7], among other negative health conditions. Further, physically active youth are likely to have better school attendance, grades, classroom behavior, and cognitive function [3,4].

Children spend a substantial amount of time at school, making it an ideal place to embed PA within the day [2]. Youth have been found to be more physically active during the school year than during the summer [8,9], and this pattern is even more pronounced for certain subgroups, such as Hispanic or Latinx youth [9]. One explanation for this may be the structured day hypothesis, which posits that the presence of structure (preplanned, segmented, and adult supervision) may regulate obesogenic behaviors, including physical inactivity and sedentary behaviors, in youth [10].

In March 2020, the World Health Organization declared COVID-19 a pandemic [11]. In response, many schools closed for the academic year 2 months earlier than usual to curb the spread of COVID-19. Students participated in web-based schools for the remainder of the school year. As a result, past research has hypothesized that overall PA would be further reduced due to school closures during the COVID-19 pandemic [12]. Therefore, the purpose of this study was to objectively measure changes in youth PA from before to during COVID-19 pandemic–related school closures.

## Methods

### Participants

Beginning in January 2020, middle school youth from an urban school district in a Midwest city were recruited to participate in a 2-arm quasi-experimental study. All students in the school district qualified for free lunches [13]. Students from 2 middle schools were recruited to participate as controls, and 2 schools were recruited to participate in an after-school PA and nutrition intervention. The intervention would have provided sports sampling programming, during which 4 sessions of sports instruction would have been provided weekly. The focus sport would have rotated every 2 weeks to provide a variety. Weekly distributions of produce kits that were designed to make at least 1 meal for a family of 5 would also have been distributed. The study activities were planned to continue throughout the remainder of the school year (May 2020) and were planned to resume in fall 2021. During the recruitment process and baseline testing and prior to intervention implementation, schools closed for spring break. Following spring break, the schools remained closed, and students did not return to in-person schools for the remainder of the academic year due to the COVID-19 pandemic. This phenomenon provided a unique opportunity to conduct our natural experimental study. Prior to school closure, baseline data collection (ie, height, weight, and demographics) was completed for 86 youths; 72% (n=62) of these youths were in the control group. Due to school constraints, enrollment for intervention students was slower compared to that for control schools. Students who enrolled in the intervention stayed after school to complete enrollment and baseline testing. During this time, no structured programming had begun in intervention schools. Due to the low intervention enrollment and low dose of the intervention that was delivered, all participants (control and intervention groups) were eligible for inclusion in our analysis.

### Instrumentation and Procedures

Each youth was provided with a Garmin Vivofit 4 (Garmin Ltd) to objectively measure PA from February to April 2020. Although it is critical to account for nonwear time in research that involves using consumer activity monitors, there is a lack of consensus on the best approaches for detecting nonwear time. Approaches vary by device manufacturer (eg, Garmin Ltd vs Fitbit LLC) and device model (eg, heart rate is sometimes used but is not measured by the Vivofit 4). Although the most common approaches have been to include all days regardless of wear time (ie, no detection of nonwear) and define valid days based on a minimum step count threshold [14], we used a more rigorous approach in this study, similar to what has been used in some Fitbit-based research [15]. Groups of ≥3 epochs (15 minutes each) with a value of 0 for maximum motion intensity were considered nonwear time. Valid wear days were defined as days for which a participant had ≥8 hours of wear time between 9 AM and 9 PM and ≥500 steps. For each participant, daily data were aggregated at the week level, with a requirement of ≥1 valid day for the week to be included in the analyses. We selected the requirement of ≥1 valid day due to the stringent criteria used for a valid day; however, the mean number of valid days per week was 5.24 (SD 2.21). Weighted weekly values for steps per day and MVPA minutes per day were calculated as follows:


([mean of weekdays × 5] + [mean of weekend days × 2]) ÷ 7

When a participant did not wear the device for ≥1 weekday and ≥1 weekend day in a given week, the weekly value was calculated as a mean of all valid wear days for the week. MVPA was measured by using the Garmin device’s automated activity detector. The device automatically measures active minutes when a user runs for at least 1 minute or walks for at least 10 consecutive minutes.

Participants with at least 1 week’s worth of data in the 4 weeks prior to school closure and at least 1 week’s worth of data in the 4 weeks after school closure were included in the data analyses. The mean numbers of pre–school closure and post–school closure weeks with valid participant data were 2.65 (SD 1.11) and 3.12 (SD 1.17), respectively. This inclusion criterion allowed us to examine multiple time points of PA behavior while also allowing for a larger sample size. After removing participants who did not meet the inclusion criteria, we had 17 participants with valid data. The school closure forced a change in syncing practices; research staff went from syncing youths’ Garmin devices weekly at schools to instructing students to sync their Garmin devices to a personal device (ie, a smartphone or tablet) at home. Many of the students did not respond to the research team’s efforts to train students on the new syncing practices, which limited the number of participants who had valid data post–school closure to 17 participants. Of these 17 participants, 16 were in the control group.

### Ethics Approval

All study procedures were approved by the University of Missouri-Kansas City Institutional Review Board (2017528).

### Data Analysis

Descriptive statistics were used to analyze demographic variables. Demographic differences between youth who were included in and excluded from the analyses were assessed by using chi-square tests. Differences in the number of steps per day and MVPA minutes per week from pre–school closure (4-week period) to post–school closure (4-week period) were assessed using 2 mixed effects models (1 for each PA dependent variable). Our models accounted for the nesting of weeks within participants and were adjusted for the number of valid weekday wear days and weekend wear days in each week. A second pair of mixed effects models, which accounted for the nesting of weeks within participants, was used to investigate differences in the number of steps per day and MVPA minutes per week across the 8 study weeks. For these models, the *study week* variable was entered as a categorical fixed effect in addition to the aforementioned covariates. Estimated sample means and CIs were calculated from the models’ results and plotted. The restricted maximum likelihood was used in all models to account for missing data. Significance levels were set at *P*<.05. All analyses were conducted with SPSS (version 25; IBM Corporation).

## Results

### Demographics

The demographics of the participating youth are presented in Table 1; demographics data are presented separately for youth who were included in and excluded from analyses and are compared for statistical differences. The participants included in the data analysis (n=17) were primarily Hispanic or Latinx (8/17, 47%) and female (10/17, 59%). All participants were in the sixth, seventh, or eighth grade and represented 3 public middle schools. The majority of families (15/17, 88%) reported incomes within low-income limits for the county [16]. No significant differences were reported between those who were included in the analyses and those who were excluded from the analyses.

**Table 1:** Demographic characteristics of participants who were included in the analyses compared to those of participants who were excluded from the analyses.

Characteristic		Participants included in the analyses (n=17), n (%)	Participants excluded from the analyses (n=69), n (%)	*P* value	
**Race and ethnicity**					.66
	Black	7 (41)	22 (32)		
	Hispanic or Latinx	8 (47)	33 (48)		
	White	2 (12)	9 (13)		
	Other or preferred not to respond	0 (0)	5 (7)		
**Gender**					.78
	Male	7 (41)	28 (41)		
	Female	10 (59)	39 (57)		
	Other or preferred not to respond	0 (0)	2 (3)		
**Household monthly income (US $)**					.72
	≤1000	4 (24)	19 (29)		
	1000-2000	4 (24)	23 (35)		
	2000-3000	5 (29)	12 (19)		
	3000-4000	2 (12)	7 (11)		
	≥4000	2 (12)	4 (6)		

### PA Findings

**Figure 1:**
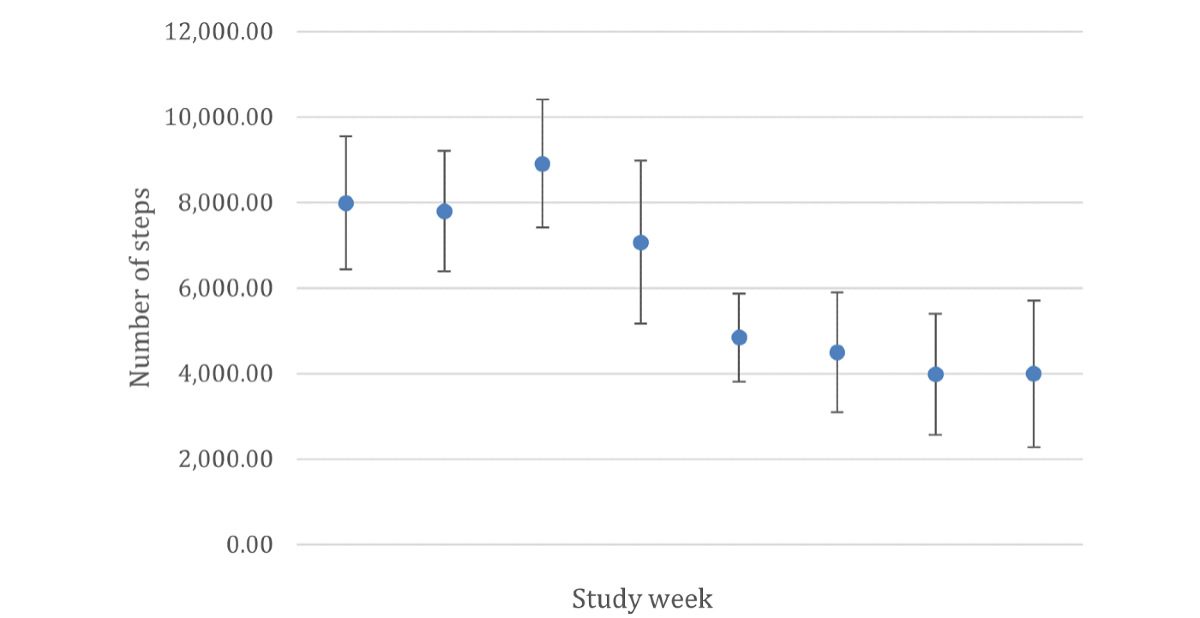
Mean number of steps per day by study week. Study weeks 1 to 4 depict pre–school closure data, and study weeks 5 to 8 depict post–school closure data. Estimated sample means are presented with error bars representing 95% CIs.

**Figure 2:**
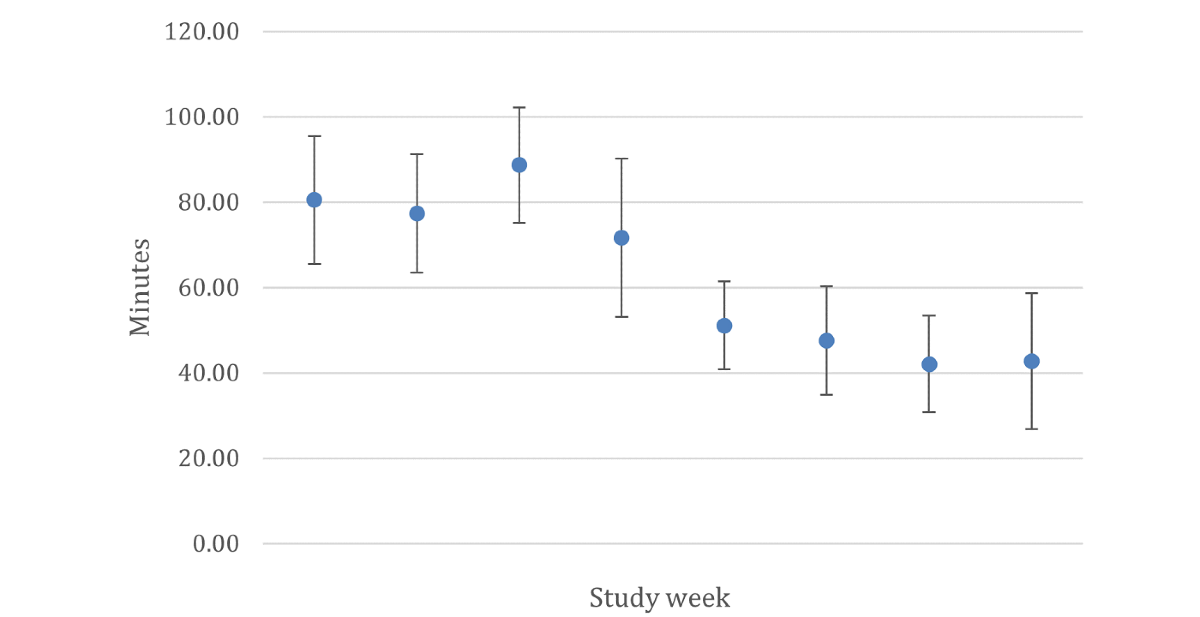
Mean moderate-to-vigorous physical activity minutes per week by study week. Study weeks 1 to 4 depict pre–school closure data, and study weeks 5 to 8 depict post–school closure data. Estimated sample means are presented with error bars representing 95% CIs.

Table 2 presents the pre– and post–school closure steps and MVPA data. Participants accumulated a mean of 8003 (SE 369.18) steps per day pre–school closure. This decreased to a mean of 4366 (SE 351.42) steps per day after school closures due to the COVID-19 pandemic, which was a significant decrease (*F*_1,6_=50.17; *P*<.001). Similarly, MVPA significantly decreased (*F*_1,6_=47.3; *P*<.001) from pre–school closure (mean 80.18, SE 3.56 minutes/week) to post–school closure (mean 46.13, SE 3.39 minutes/week). Figures 1 and 2 show that the number of steps per day and MVPA minutes per week were fairly consistent across the first 4 study weeks; these decreased during the fifth study week, which coincided with spring break, and remained low during the school closure.

**Table 2:** Changes in the number of steps and physical activity from pre–school closure to post–school closure (n=17).

Characteristic	Pre–school closure, mean (SE)	Post–school closure, mean (SE)	Percent change	*F* test (*df*)	*P* value
Number of steps per day	8003 (369.18)	4366 (351.42)	−45.4	50.17 (1,6)	<.001
MVPA^a^ minutes per week	80.18 (3.56)	46.13 (3.39)	−42.5	47.30 (1,6)	<.001

^a^MVPA: moderate-to-vigorous physical activity.

## Discussion

### Principal Findings

This study aimed to objectively assess the change in youth PA from before to during school closures due to the COVID-19 pandemic. Overall, the sample was small and was comprised of mostly racial and ethnic minority students (15/17, 88%). Similar to findings from a scoping review of other studies conducted in the first year of the COVID-19 pandemic [17], a significant decrease in objectively measured PA was recorded for the participants (*P*<.001). The findings from this study add to the previous literature by providing longitudinal data that spans pre– and post–school closure time points, as the majority of previous studies were cross-sectional and provided no comparison data for PA prior to the pandemic. Moreover, this is the only study, to our knowledge, to use pre– and post–school closure device-based measurements for youth in the United States. The large decrease in PA that we observed in our study may be partially explained by the decreases in PA among racial and ethnic minority groups during school breaks, which are more significant than those among White youth [9]. It appears that the structure and opportunities for movement provided during a web-based school day, at least during the early delivery of web-based schooling, were not sufficient for maintaining PA, as hypothesized in the structured day hypothesis [10].

Childhood obesity and inactivity continue to plague youth in the United States. PA tends to decline during middle school years—a time when recess and physical education requirements often decrease. Additionally, COVID-19 has exacerbated previously identified declines in PA. Schools are still an ideal place for implementing policies and interventions that improve health behaviors because of the significant time spent at school by most youth [2], but new strategies may be needed, particularly when schools are conducting web-based learning. Schools should consider implementing active learning and encouraging movement between classes. Middle school administrators should examine policies regarding physical education requirements and aim to achieve the recommended 225 minutes per week of physical education instruction [18]. Before- and after-school activities are highlighted as key strategies for supplementing youth PA levels [19]. In summary, middle schools need to expand their offerings for increasing PA as students return to in-person learning. This will not only help students achieve prepandemic levels of PA but also help them meet the recommended 60 minutes of PA every day [2].

Although schools have traditionally been a setting for youth to obtain the majority of their recommended PA, the effects of the COVID-19 pandemic suggest that other settings may need to foster a greater proportion of youths’ PA. Further, while youth spend more time at home, parents will likely play a more significant role in encouraging PA during the pandemic. However, parents have indicated that they need resources to help them support their child’s PA [20]. Web-based, after-school PA programming may be one strategy for increasing PA. Such programming should be tailored to students in middle school, where the activities are fun, involve friends, and include some competition [21]. Lastly, a variety of outdoor activities should be encouraged (eg, nature walks, bike rides, games, etc).

### Strengths and Limitations

This study has several strengths. One is its natural quasi-experimental design. Additionally, this study is strengthened by its use of objective measures of PA and offers longitudinal time points that demonstrate the change in PA behavior from before to during the school closures resulting from the COVID-19 pandemic. By using objective measures of PA, participant recall biases were eliminated. Racial and ethnic minority groups are often left out of PA research; this study is strengthened by the participation rates of these groups.

This study also has limitations. The sample for this study was small, and the proportion of participants included in the analyses was also small. There were several days with missing data, which required us to compute weekly means for the number of steps taken. Additionally, we were forced to change the syncing procedures for the Garmin accelerometers due to the school closure. Before the school closure, the research team synced participants’ accelerometers at school weekly. After the school closure, we needed youth to register their accelerometers to a personal device, which posed significant issues for consistent data collection, as reflected by our lower number of valid weeks post–school closure. Lastly, social distancing and other precautionary measures that were enforced upon the youth may have impacted other opportunities for PA. Further research with larger sample sizes is needed to confirm our results. Many schools continue to offer web-based learning, and they should be studied to determine if the low PA trends have held consistent.

### Conclusions

This study revealed a significant decrease in PA among middle school student participants after the COVID-19–related school closures. As schools remain web-based or continue to have periods of web-based learning, it should be a priority for schools to incorporate regular PA within the school day. Schools and partnering organizations should provide extracurricular PA opportunities, even if they are provided on the web or through other noncontact formats.

## Abbreviations

MVPA: moderate-to-vigorous physical activity
PA: physical activity
